# First-in-Man Demonstration of Direct Endothelin-Mediated Natriuresis and Diuresis

**DOI:** 10.1161/HYPERTENSIONAHA.116.08832

**Published:** 2017-05-30

**Authors:** Robert W. Hunter, Rebecca Moorhouse, Tariq E. Farrah, Iain M. MacIntyre, Takae Asai, Peter J. Gallacher, Debbie Kerr, Vanessa Melville, Alicja Czopek, Emma E. Morrison, Jess R. Ivy, James W. Dear, Matthew A. Bailey, Jane Goddard, David J. Webb, Neeraj Dhaun

**Affiliations:** From the British Heart Foundation Centre of Research Excellence and The Queen’s Medical Research Institute, University of Edinburgh, United Kingdom.

**Keywords:** diuresis, endothelin, hypertension, kidney, natriuresis

## Abstract

Endothelin (ET) receptor antagonists are potentially novel therapeutic agents in chronic kidney disease and resistant hypertension, but their use is complicated by sodium and water retention. In animal studies, this side effect arises from ET_B_ receptor blockade in the renal tubule. Previous attempts to determine whether this mechanism operates in humans have been confounded by the hemodynamic consequences of ET receptor stimulation/blockade. We aimed to determine the effects of ET signaling on salt transport in the human nephron by administering subpressor doses of the ET-1 precursor, big ET-1. We conducted a 2-phase randomized, double-blind, placebo-controlled crossover study in 10 healthy volunteers. After sodium restriction, subjects received either intravenous placebo or big ET-1, in escalating dose (≤300 pmol/min). This increased plasma concentration and urinary excretion of ET-1. Big ET-1 reduced heart rate (≈8 beats/min) but did not otherwise affect systemic hemodynamics or glomerular filtration rate. Big ET-1 increased the fractional excretion of sodium (from 0.5 to 1.0%). It also increased free water clearance and tended to increase the abundance of the sodium–potassium–chloride cotransporter (NKCC2) in urinary extracellular vesicles. Our protocol induced modest increases in circulating and urinary ET-1. Sodium and water excretion increased in the absence of significant hemodynamic perturbation, supporting a direct action of ET-1 on the renal tubule. Our data also suggest that sodium reabsorption is stimulated by ET-1 in the thick ascending limb and suppressed in the distal renal tubule. Fluid retention associated with ET receptor antagonist therapy may be circumvented by coprescribing potassium-sparing diuretics.

## Abstract 2

Supplemental Digital Content is available in the text.

The endothelin (ET) peptide family was described by Yanagisawa et al^1^ in 1988. They form an intricate signaling system, in which 3 mature peptides (ET-1, ET-2, and ET-3) interact with 2 receptors (ET_A_ and ET_B_).^2,3^ ET-1 is the main vascular endothelial species and the most powerful vasoconstrictor. It is generated from a precursor peptide, big ET-1, through proteolytic cleavage by 2 ET-converting enzymes (ECE-1 and ECE-2, which exist in several isoforms) or other proteases.

The kidney is both a source and site of action of ET-1. Indeed, the renal medulla contains the highest concentration of immunoreactive ET-1 in the body.^4^ Significant amounts of ET-1 are detectable in most renal cell types, probably acting as a paracrine/autocrine regulator of renal and intrarenal blood flow, glomerular hemodynamics, and sodium and water transport.^4^ ET_A_ receptors are situated on vascular smooth muscle cells where they promote vasoconstriction and are thought to mediate many of the pathological effects of ET-1.^5^ In the kidney, ET_B_ receptors are expressed by the vascular endothelium, vascular smooth muscle, and tubular epithelial cells along the length of the nephron—with a particularly high density in the medullary collecting ducts.^3^ In vascular endothelium, ET_B_ receptor activation promotes vasodilation.^2^ In the renal tubule, preclinical data suggest that ET_B_ receptors stimulate natriuresis and diuresis.^3,6^ To date, this action has not been demonstrated in man.

Understanding the effect of ET signaling on renal salt and water transport is important because the system makes an attractive target for novel therapies in disorders of fluid-electrolyte homeostasis. An inability to appropriately excrete salt and water is a feature of many common conditions including chronic kidney disease (CKD), chronic liver disease, congestive heart failure, and salt-sensitive hypertension. Importantly, salt and water retention is associated with both acute and chronic morbidity and mortality.^7,8^ ET receptor antagonists, a novel class of drug currently licensed for the treatment of pulmonary arterial hypertension and scleroderma digital ulcers,^2^ are being investigated in a range of clinical conditions including diabetic nephropathy, heart failure, and resistant hypertension.^2^ However, salt and water retention is a common side effect of these agents and has led to the premature termination of a phase 3 clinical trial.^9^

It has been hypothesized that blockade of ET_A_ receptors confers clinical benefit (reducing blood pressure [BP], proteinuria, and renal inflammation), whereas off-target blockade of ET_B_ in the renal tubule induces deleterious sodium and water retention.^10^ This hypothesis is supported by data from animal models, in which low doses of ET-1 promote natriuresis and diuresis in the absence of significant hemodynamic change, by stimulating ET_B_ receptors in the renal tubule.^11^ However, a direct natriuretic action of ET-1 has not yet been demonstrated in man. Previous studies have been confounded by the changes in systemic hemodynamics induced by ET receptor agonism and antagonism. For example, the administration of exogenous ET-1 produces profound retention of salt and water accompanied by systemic and renal vasoconstriction,^12,13^ whereas ET_A_ blockade in CKD induces a natriuresis and an increase in renal blood flow.^14^ It is not possible to easily differentiate the indirect effect of any hemodynamic changes on renal salt and water excretion from direct effects on renal tubular cell function.

The aim of this study was to demonstrate a direct natriuretic effect of ET receptor activation in man. We administered incremental doses of intravenous big ET-1 to healthy volunteers. We used big ET-1 (as opposed to ET-1), as this would more closely reflect normal physiology, with ET-1 being generated only in those tissues expressing ECE. We hypothesized that big ET-1 would lead to a gradual increase in natriuresis and free water clearance (FWC) in the absence of significant hemodynamic changes.

## Methods

### Subjects

This was a 2-phase randomized, double-blind, placebo-controlled crossover study in 10 healthy volunteers. The study was performed with the approval of the local research ethics committee and the written informed consent of each subject. The investigations conformed to the principles outlined in the Declaration of Helsinki.

Male and female subjects were recruited from the community between September 2006 and January 2007. To be eligible for inclusion, subjects had to be between 18 and 80 years of age with no medical history documented by their primary care physician and prescribed no regular medications. Other inclusion criteria were a body mass index <30 kg/m^2^, BP <140/90 mm Hg, normal biochemical parameters, and a clear urinalysis.

### Study Protocol

Subjects were asked to adhere to a standardized diet (avoiding high salt–containing foods) for 3 days before each study day and to complete two 24-hour urine collections during this time, the second completing on the morning of the study. These were to assess daily sodium intake. Subjects abstained from alcohol, caffeine, and smoking for 48 hours before each study phase and, apart from a light breakfast on the study day, remained fasted throughout each study phase.

All studies were performed at the same time of day in a quiet, temperature-controlled room. The protocol for each study day is summarized in Figure S1 in the online-only Data Supplement. In brief, after an initial 500 mL bolus of 5% dextrose to initiate diuresis, a maintenance infusion (300 mL/h) continued throughout the study. After a 1.5-hour equilibration period, baseline measurements were made over the next half hour, after which placebo or big ET-1 (Clinalfa) were administered. Systemic hemodynamic and renal responses were then followed for a period of 2.5 hours after the infusions were commenced. BP, cardiac output, cardiac index, and heart rate were recorded throughout the study by well-validated noninvasive automated techniques^15,16^ every 15 minutes, and urine was collected every 30 minutes by spontaneous voiding while standing. The doses of big ET-1 used here were based on previous studies.^17,18^

Alongside systemic hemodynamic and renal responses, pulse wave velocity, the gold standard for measurement of arterial stiffness,^19^ was measured every 30 minutes by the foot-to-foot wave velocity method using the SphygmoCor system (SphygmoCor Mx, AtCor Medical, Sydney, Australia; version 6.31), in which a high-fidelity micromanometer (SPC-301; Millar Instruments, TX) was used to determine carotid–femoral pulse wave velocity.

### Sample Collection and Analysis

Samples of venous blood were collected every 30 minutes into EDTA tubes (Sarstedt) for measurement of plasma ET-1 and osmolality and into plain tubes (Sarstedt) for serum creatinine and sodium. Additionally, after measuring urine volume, 20 mL aliquots from each voiding were collected into plain tubes for the measurement of urinary creatinine, sodium, and osmolality. For urine ET-1, a 20 mL aliquot of urine was collected into plain tubes with 2.5 mL of 50% acetic acid. Blood samples were centrifuged immediately at 2500*g* for 20 minutes at 4°C. All samples were stored at −80°C until analysis.

Plasma and urine ET-1 were determined by ELISA (R&D systems). The mean recovery of ET-1 was >95%. The intra- and interassay variations were 4% and 6%, respectively. The cross-reactivity of the assay was 23% for ET-2, 0.5% for ET-3, and there was no cross-reactivity with big ET-1. Plasma and urine sodium concentrations were measured using an ion-selective electrode. Urine calcium concentration was measured by flame photometry (BWB Technologies UK). Plasma and serum osmolality was measured by freezing point depression using a standard osmometer. Plasma vasopressin concentration was determined by ELISA (Enzo Labs). The concentration of total urinary nitrite and nitrate (NOx) was measured by colorimetric assay (780001; Cayman Chemicals, Ann Arbor, MI).

### Preparation of Urinary Extracellular Vesicles

In 5 subjects, urinary extracellular vesicles were prepared by ultracentrifugation as previously described.^20,21^ Urine samples were taken from the collection made between 90 and 120 minutes after treatment with placebo or big ET-1. This was the time point at which maximal natriuresis and FWC were seen.

### Immunoblotting

Immunoblot analyses were conducted with the experimenter blinded to the treatment received. The primary antibodies were rabbit anti-AQP2 (AB3274; Millipore; 1:600), sheep anti-NKCC2 (DSTT Dundee; 1:10 000), and rabbit anti-NCC (AB3553; Millipore; 1:1000); the secondary antibodies were horseradish peroxidase–conjugated goat antirabbit Ig (sc-2301; Santa-Cruz; 1:2000) and horseradish peroxidase–conjugated donkey antisheep Ig (A3415; Sigma; 1:20 000). Immunoblot analyses were conducted with the experimenter blinded to the treatment received (hence, the lack of systematic lane order). In the densitometry analysis, band density was divided by the time taken to collect the urine sample used for urinary extracellular vesicle preparation so that each result represents the abundance of antigen excreted per unit time.

### Data Analysis

Data were stored and analyzed in Graph Pad Prism, version 6.0 (GraphPad Software, Inc, San Diego, CA). Systolic and diastolic BP at each time point was calculated as the mean of 2 recordings. Mean arterial pressure was calculated as diastolic BP+1/3 pulse pressure. Bioimpedance data at each time point were calculated as the mean of 4 recordings, each the average of 15 consecutive heart beats. Data were corrected for body surface area to give cardiac index, for direct comparison between subjects. Systemic vascular resistance index was calculated by dividing mean arterial pressure by cardiac index and expressed in dyne/s/m^2^/cm^5^/100. Urinary sodium excretion (UNaV) and ET-1 excretion were calculated as (urinary sodium×urinary flow rate) and (urinary ET-1×urinary flow rate), respectively. The fractional excretion of sodium (FeNa) and ET-1 (FeET-1) were calculated as ([urine sodium/serum sodium×serum creatinine/urine creatinine]×100)% and ([urine ET-1/plasma ET-1×serum creatinine/urine creatinine]×100)%, respectively. FWC was calculated as (urine flow rate×1-urine osmolality/plasma osmolality).

### Statistical Analysis

Baseline hemodynamic data were calculated as the mean of the 2 time points that immediately preceded administration of the study drug. For urine data, only one baseline measurement was used immediately before drug dosing. Hemodynamic and urine results are expressed as mean±SEM change from baseline for drug and placebo. Statistical analysis was performed on untransformed data. Responses were examined by repeated-measures ANOVA, and Bonferroni correction was used to assess significance at specific time points. Statistical significance was taken at the 5% level.

## Results

All 10 subjects fully completed the placebo and big ET-1 phases of the study without adverse events. All subjects had similar baseline 24-hour urinary sodium excretion on each study day. Subject demographics and baseline parameters are shown in Table.

**Table.**
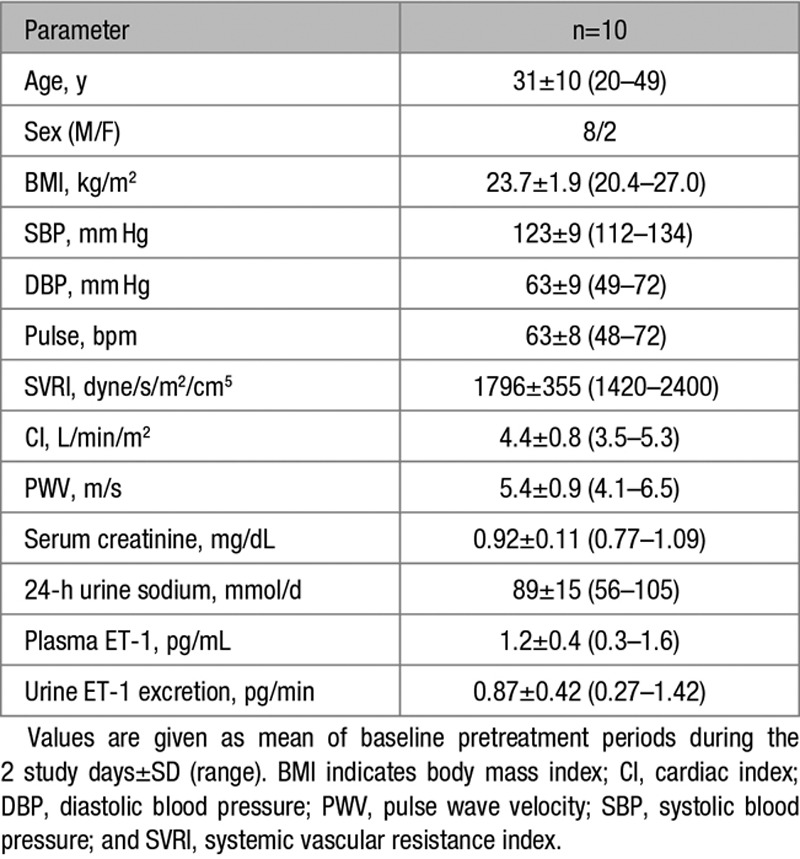
Baseline Study Participant Data

### Plasma and Urinary ET-1

**Figure 1.**
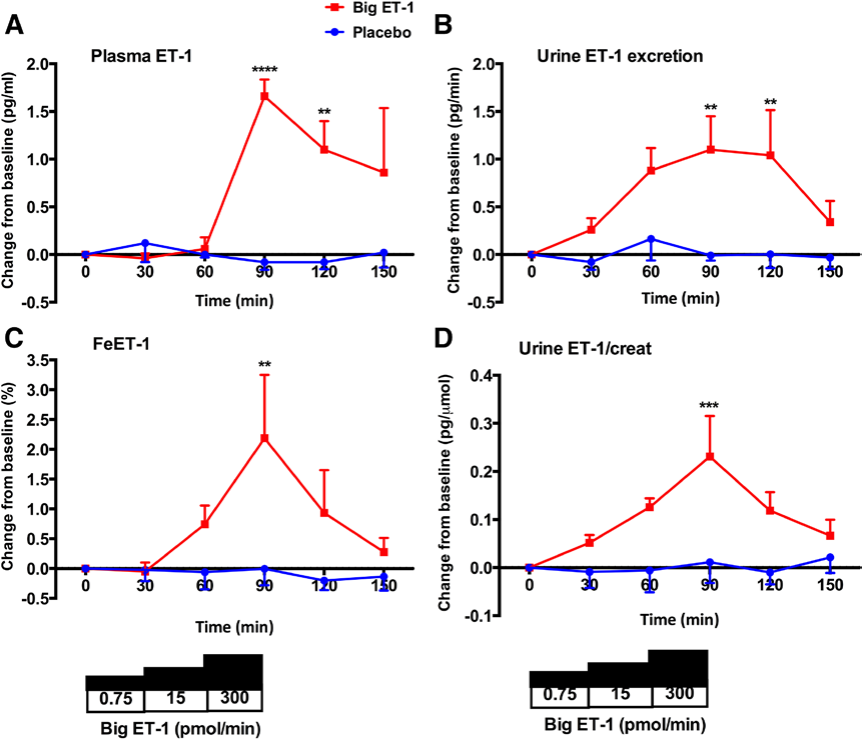
Change in plasma and urinary endothelin-1 (ET-1). Change from baseline±SEM in plasma ET-1 (**A**), urinary ET-1 excretion (**B**), fractional excretion of ET-1 (FeET-1; **C**), and urine ET-1/creatinine after treatment with placebo (blue line) and big ET-1 (red line). **P<0.01, ***P<0.001, and ****P<0.0001 for placebo vs big ET-1 (ANOVA plus Bonferroni correction for significance at specific time points).

Placebo was not associated with any changes in plasma or urinary ET-1 (Figure 1A through 1D). Infusion of big ET-1 led to a ≈1.5-fold increase in circulating ET-1 but only after the highest dose (Figure 1A). Plasma ET-1 gradually fell after the infusion of big ET-1 stopped. In parallel with this increase in circulating ET-1, there was a gradual ≈2.5-fold rise in urinary ET-1 excretion from 0.78 to 1.97 pg/min (Figure 1B). There were 4-fold increases in fractional excretion of ET-1 (FeET-1) from 0.6% to 2.4% (Figure 1C) and urine ET-1/creatinine from 0.05 to 0.20 pg/µmol (Figure 1D).

### Systemic Hemodynamics

**Figure 2.**
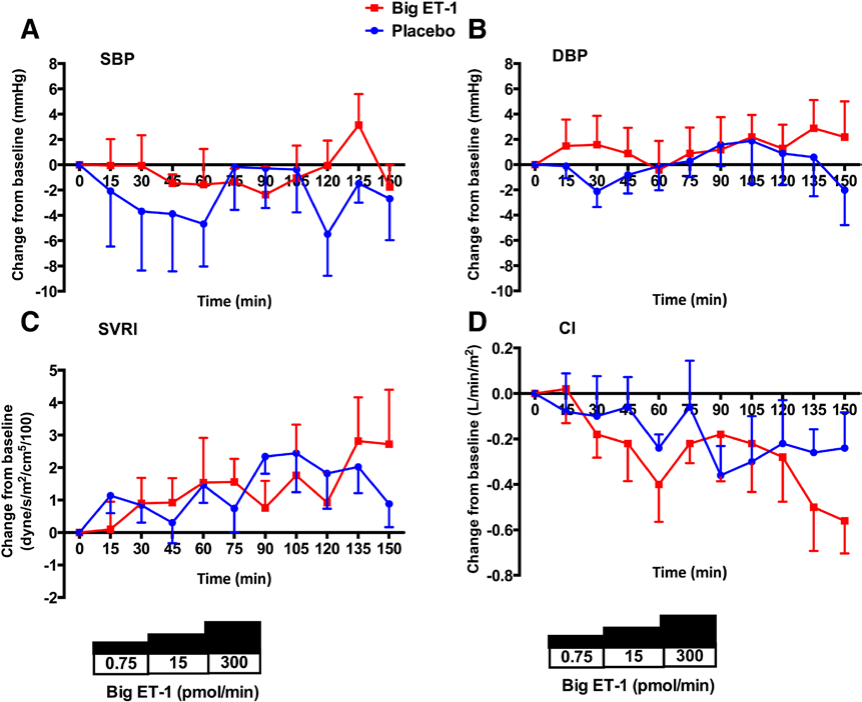
Changes in systemic hemodynamics. Change from baseline±SEM in systolic BP (SBP; **A**), diastolic BP (DBP; **B**), systemic vascular resistance index (SVRI; **C**), and cardiac index (CI; **D**) after treatment with placebo (blue line) and big endothelin-1 (ET-1; red line).

**Figure 3.**
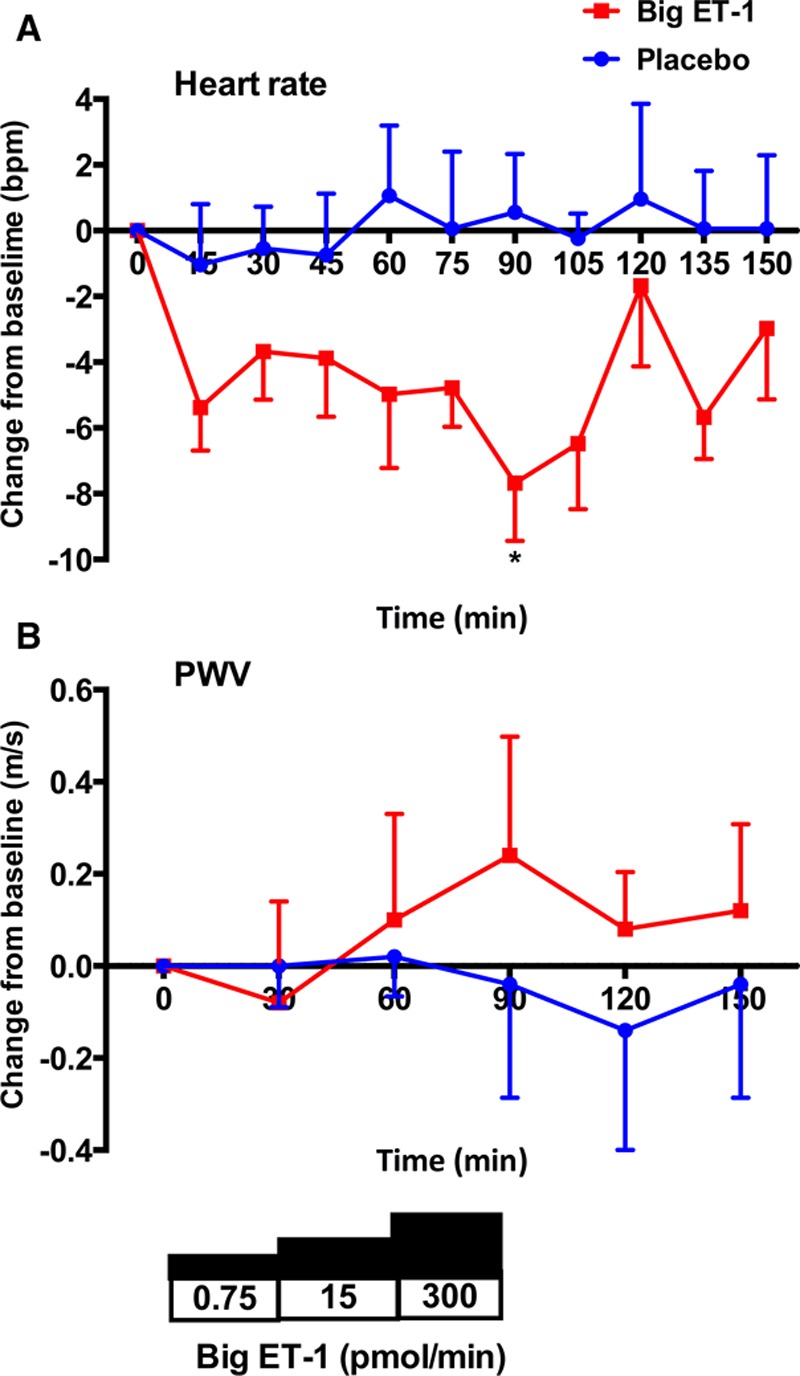
Changes in heart rate and arterial stiffness. Change from baseline±SEM in heart rate (**A**) and pulse wave velocity (PWV; **B**) after treatment with placebo (blue line) and big endothelin-1 (ET-1; red line). *P<0.05 for placebo vs big ET-1 (ANOVA plus Bonferroni correction for significance at specific time points).

Neither placebo nor big ET-1, at any of the 3 doses infused, were associated with changes in systolic or diastolic BP, systemic vascular resistance index, or cardiac index (Figure 2A through 2D). Whereas placebo was not associated in any change in heart rate, infusion of big ET-1 led to an early and sustained fall in heart rate over the course of the study—a maximal fall of ≈8 bpm (Figure 3A). In keeping with the lack of change in BP, there was no change in pulse wave velocity with either placebo or big ET-1 (Figure 3B).

### Renal Responses

**Figure 4.**
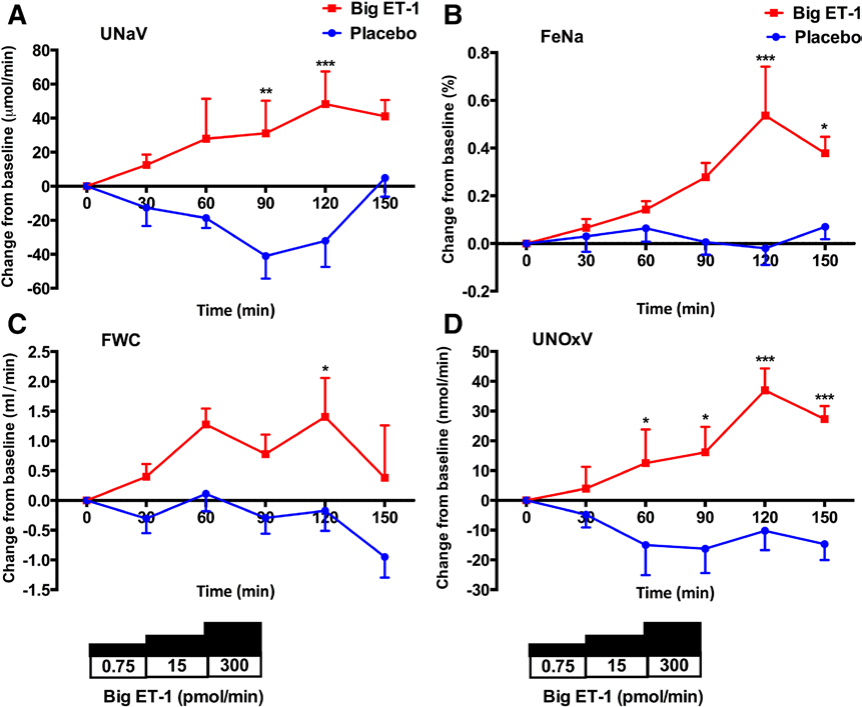
Changes in renal responses. Change from baseline±SEM in urinary sodium clearance (UNaV; **A**), fractional excretion of sodium (FeNa; **B**), free water clearance (FWC; **C**), and urinary excretion of NO metabolites (UNOxV; **D**) after treatment with placebo (blue line) and big endothelin-1 (ET-1; red line). *P<0.05, **P<0.01, and ****P<0.0001 for placebo vs big ET-1 (ANOVA plus Bonferroni correction for significance at specific time points).

Compared with placebo, big ET-1 infusion was associated with a gradual increase in UNaV (Figure 4A). This was maximal at 120 minutes, 30 minutes after the completion of the highest dose of big ET-1, and equated to ≈40 µmol/min. There was no change in creatinine clearance, plasma concentration of cystatin C, or potassium excretion with either placebo or big ET-1 over the time course of the study (Figure S2A though S2C).

Because UNaV may be affected by more subtle changes in intrarenal hemodynamics that may not be reflected by the creatinine clearance, we calculated FeNa as a measure of tubular sodium handling. Placebo was not associated with any change in FeNa over the time course of the study. By comparison, big ET-1 led to a marked natriuresis with a doubling of FeNa from 0.5% to 1.0% (Figure 4B). Placebo did not affect FWC. However, FWC did increase with big ET-1 but only after the highest dose—an increase from 4.1 to 5.5 mL/min (Figure 4C). Interestingly, there was a positive correlation between the maximal change in FeET-1 and the maximal increase in FWC (*r*=0.83; *P*=0.003) such that those subjects showing the greatest increase in renal ET-1 production had the greater increase in FWC (Figure S3A). There was a trend for this association between FeET-1 and FeNa (*r*=0.64; *P*=0.05; Figure S3B). The summed urinary excretion of nitrates and nitrites (UNOxV) increased after the administration of big ET-1 (Figure 4D), compatible with increased renal production of NO.

**Figure 5.**
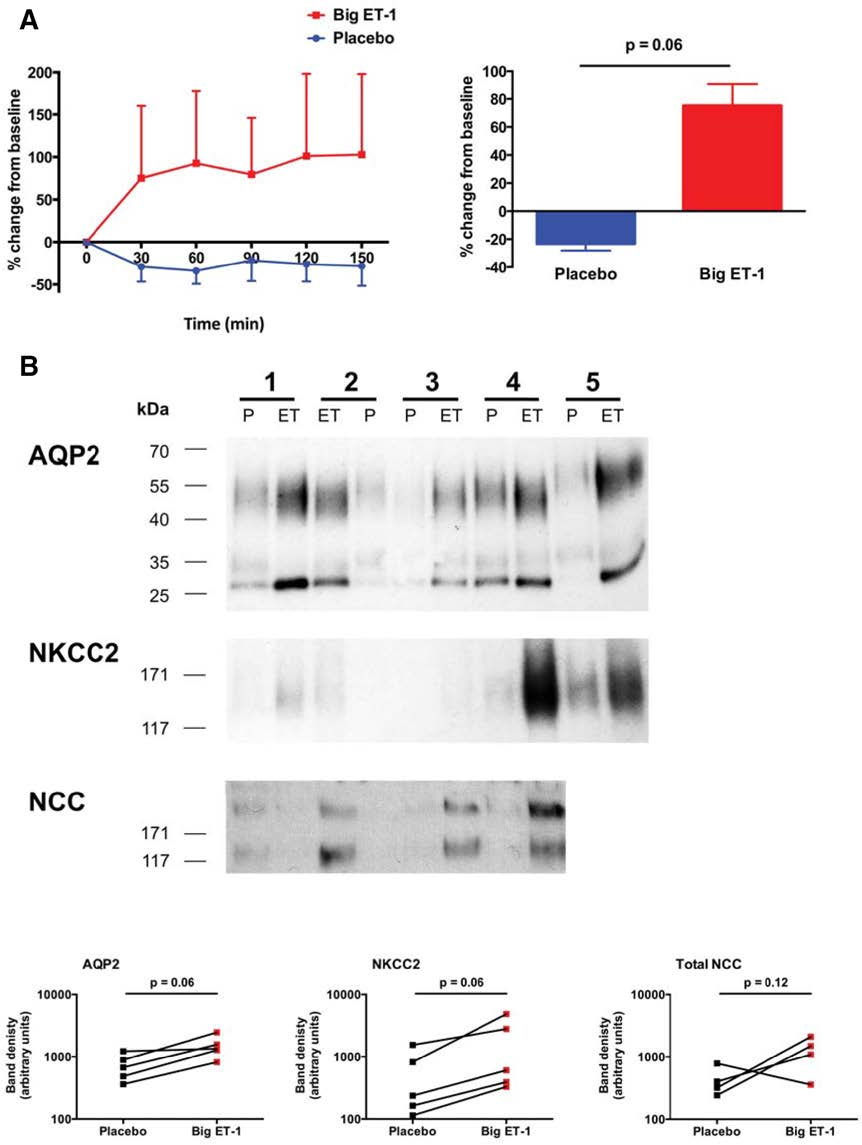
Molecular mechanisms regulating free water clearance. Change from baseline±SEM in plasma concentration of vasopressin (**A**). Effect of big endothelin-1 (ET-1) on the abundance of AQP2, NKCC2, and sodium-chloride cotransporter (NCC) in urinary extracellular vesicle (uEVs), assessed by immunoblot (**B**). For AQP2, bands were detected at 28–35 and 40–55 kDa, corresponding to the nonglycosylated and glycosylated forms, respectively. Urine samples from subject number 5 were not available in sufficient quantity to prepare uEVs for the NCC blot.

In a subset of subjects (n=5), we explored the molecular mechanisms responsible for the increased FWC induced by big ET-1. Big ET-1 tended to increase the plasma concentration of vasopressin (Figure 5A). There was also a trend to an increase in the abundance of aquaporin 2 (AQP2) and the bumetanide-sensitive sodium–potassium–chloride cotransporter (NKCC2; Figure 5B). There was no significant difference in the abundance of the thiazide-sensitive sodium-chloride cotransporter (Figure 5B).

Additionally, we performed a more detailed clearance analysis in this subset. Thus, subjects were maintained in hypotonic diuresis, and in this context, the fractional delivery of sodium to the thick ascending limb (TALH) can be estimated by (*C*H_2_O+*C*Na)/100 mL glomerular filtration rate and the fraction of this sodium load that is reabsorbed in the diluting segment (approximating to the TALH) by (*C*H_2_O/[*C*H_2_O+*C*Na]×100)%.^22,23^ The effects of big ET-1 on these parameters are shown in Figure S4. Big ET-1 increased the measure of sodium reabsorption in the diluting segment but had no effect on the estimate of fractional sodium delivery to the TALH. Urinary calcium excretion was lower after big ET-1, but this did not reach significance: 0.103±0.006 versus 0.083±0.009 mg per minute (placebo versus big ET-1; *P*=0.078).

Finally, because we were unable to discriminate between ET_A_- and ET_B_-mediated effects in the current study and to highlight the clinical importance of our findings, we analyzed FWC in one of our previous studies.^24^ Here, in both healthy volunteers and those with CKD, we have demonstrated that selective ET_B_ receptor antagonism, but not selective ET_A_ or mixed ET_A/B_ blockade, diminishes the capacity to clear free water (Figure S5).

## Discussion

In this study, we have shown, for the first time in man, that big ET-1 stimulates renal salt excretion. This is independent of changes in systemic hemodynamics, arterial stiffness, and estimated glomerular filtration rate and so most likely reflects a direct action of ET-1 on the renal tubule.

### Effects of Big ET-1 Are Likely Mediated Through Its Intrarenal Conversion to ET-1

It is unlikely that the effects seen in our study are because of big ET-1 directly, as radioligand-binding studies have established that big ET-1 does not bind to either ET_A_ or ET_B_ receptors, nor does it have any other recognized binding site.^25^ Furthermore, the hemodynamic effects seen after big ET-1 infusion in man fully depend on its conversion to ET-1.^26^ In the current study, infusion of big ET-1 led to a large rise in circulating ET-1 in keeping with vascular conversion as seen previously.^27^ Furthermore, given that once formed ET-1 is likely to bind to its receptors from which it dissociates slowly, plasma ET-1 concentration is probably an underestimate of its production. The exact site of the vascular conversion of big ET-1 to ET-1 remains unclear but is unlikely to be in whole human blood^28^ or in the vascular endothelium.^29^ The extracellular surface of the smooth muscle cell has been identified as a plausible location for this conversion.^30^

It seems likely that the increase in urinary ET-1 excretion was because of the intrarenal conversion of big ET-1 to ET-1 for 2 reasons. First, this can be predicted from the existing literature. Isotope studies in animals^31^ and clearance studies in man^32^ have shown that ET-1 in the urine is likely to be generated within the kidney (rather than being filtered or secreted into the urinary space from the plasma). ECE isoforms are expressed in the renal microvascular and the tubular epithelium.^3,33^ Second, urine ET-1 excretion (measured as net excretion or as fractional excretion of the filtered load) increased before any detectable rise in circulating ET-1 (ie, when big ET-1 was infused at lower doses). This short time course also suggests that ET-1 was likely to be generated from the intrarenal conversion of big ET-1 to mature peptide rather than de novo generation. Our data are consistent with a previous smaller study in healthy man that suggested an increase in renal ET-1 production after big ET-1 infusion.^34^

### Lack of Significant Hemodynamic Effect

Importantly, big ET-1 did not affect systemic hemodynamics at any of the doses given. There was no detectable change in systolic or diastolic BP. There was no evidence of systemic vasoconstriction, given the lack of change in systemic vascular resistance or cardiac output. We did observe a fall in heart rate, the magnitude of which was similar to that seen in earlier studies where big ET-1 has been infused in human subjects.^18,34^ This is unlikely to be a baroreceptor reflex because BP remained constant. Thus, this negative chronotropy likely reflects a direct effect of ET-1 on the heart, an effect previously reported in preclinical studies.^35^ We observed no change in creatinine clearance or plasma cystatin C, both estimates of glomerular filtration rate, over the time course of the study, suggesting that there was no significant change in renal hemodynamics at the doses of big ET-1 used. For comparison, a previous study that infused big ET-1 into healthy humans and found significant changes in systemic and renal hemodynamics used a dose that was ≈2-fold greater than the highest dose used here (≈600 pmol given intravenously over 20 minutes compared with 300 pmol given over 30 minutes here).^34^ Others have administered a similar dose of big ET-1 to that used in the current study to patients with end-stage renal disease, and this did indeed increase BP (and reduce splanchnic blood flow), but this likely reflects the fact that patients with renal disease have an activated ET system and are more sensitive to its effects.^4,24^

However, we cannot exclude regional changes in renal cortical or medullary blood flow (which are not readily assessable in man). Such changes are recognized after infusion of ET-1 in animals^36,37^ and may affect tubular handling of salt and water. Indeed, we found that the excretion of NO metabolites was increased after big ET-1. In rodent models, ET-1 has been shown to increase renal NO production (through its action on ET_B_ receptors in the collecting duct),^38^ which elicits diuresis and natriuresis.^39,40^ These effects of NO may be mediated, in part, by changes in regional blood flow within the kidney.^41^

### Natriuretic Effect of Big ET-1

As hypothesized, big ET-1 infusion led to a significant natriuresis as shown by the increases in both UNaV and FeNa of ≈40% from baseline. If this was maintained over a 24-hour period, it would amount to ≈60 mmol of sodium excreted in the urine and is broadly equivalent to the natriuresis elicited by a single 25 mg dose of spironolactone.^42^ Our subjects were healthy and relatively sodium restricted (with a sodium intake of ≈90 mmol Na or ≈5 g NaCl per day, ≈50% of that contained in a standard Western diet); it is unclear whether the natriuretic effect of ET-1 would be different on a high-salt diet or in patient populations in which there is activation of the renin–angiotensin–aldosterone system, such as in congestive heart failure, CKD, or liver cirrhosis. This would be of particular interest, because maximizing renal salt and water excretion is an important focus of clinical management in these patient groups.

In animal models, ET-1 inhibits sodium reabsorption along the length of the renal tubule.^3,43^ Our data strongly suggest that the natriuretic effect of big ET-1 is not localized to the TALH. Indeed, clearance data (*C*H_2_O/[*C*H_2_O+*C*Na]) point to increased sodium reabsorption in this segment, consistent with the ≈35% increase in FWC and the trend to reduced urinary calcium excretion.^22,23^

These functional data are supported by our molecular data showing that the abundance of NKCC2 on urinary extracellular vesicles was increased by big ET-1 (and furthermore that the observed increase in AQP2 expression occurred in a direction that would oppose free water clearance). Enhanced transport in the TALH sits at odds with the established literature; ET-1 has been consistently shown to inhibit NKCC2 activity (via ET_B_ receptors acting through NO-dependent and NO-independent pathways).^3,44^ This apparent conflict may be explained by our finding that big ET-1 increased the concentration of circulating vasopressin (another established ET-1 effect).^45^ Vasopressin increases the renal expression of both NKCC2 and AQP2, and we speculate that this indirect effect of big ET-1 over-rides any direct effect of ET receptor activation in the TALH. As overall FeNa increased, if sodium reabsorption was no different in the proximal tubule or distal convoluted tubule and stimulated in the TALH, then sodium reabsorption must have been suppressed elsewhere in the nephron (ie, connecting tubule or collecting ducts). We attempted to assess the abundance of ENaC subunits in urinary vesicles but were unable to generate detectable bands on Western blot (presumably because of the low abundance of antigen in urine samples that were rendered dilute by the water-loading protocol).

### ET_B_ Receptors Are Likely to Mediate Aquaresis and Natriuresis

Preclinical studies have demonstrated that big ET-1^46^ and ET-1^3^ promote natriuresis and diuresis through an ET_B_ receptor-mediated, NO-dependent, mechanism. In the present study, we did not attempt to directly differentiate between ET_A_- and ET_B_-mediated effects. However, in a separate cohort of healthy volunteers and patients with CKD, we have demonstrated that ET_B_ blockade diminishes the capacity to clear free water. Thus, the results of our present study are consistent with big ET-1 acting (after conversion to ET-1) on ET_B_ receptors to increase free water clearance. Furthermore, our finding of increased excretion of NO metabolites supports an ET_B_-mediated effect.

### Perspectives

In healthy volunteers, subpressor doses of big ET-1 induced natriuresis and aquaresis, effects likely mediated through renal tubular ET_B_ receptors (after intrarenal conversion to mature ET-1). Our clearance and molecular data suggest that sodium reabsorption was stimulated by ET-1 in the TALH but inhibited in distal nephron segments. Taken together with the preclinical literature, our data support the potential use of highly selective ET_A_ receptor antagonists in clinical conditions associated with salt and water retention. In line with this, preclinical studies in congestive heart failure supported a selective ET_A_-blocking approach.^5^ Long-term clinical studies, however, have been disappointing. However, all have used nonselective antagonists or modestly ET_A_-selective antagonists at doses that probably block the ET_B_ receptor. Therefore, it may well be that a truly ET_A_-selective approach has not yet been studied. Fortunately, studies using a selective approach in CKD have been encouraging,^14,47^ and the outcomes of a large phase 3 study (SONAR [Study of Diabetic Nephropathy With Atrasentan]) are eagerly awaited.^48^ As an alternative to selective ET receptor blockade, our data provide a rationale for testing potassium-sparing diuretics (rather than loop diuretics) in an attempt to ameliorate the fluid retention associated with ET receptor antagonists. Although this strategy has not, to our knowledge, been tested prospectively, a post hoc analysis of the ARIES trial (Ambrisentan in Pulmonary Arterial Hypertension, Randomized, Double-Blind, Placebo-Controlled, Multicenter, Efficacy Studies) found that coprescription of spironolactone with ambrisentan was associated with better outcomes in pulmonary arterial hypertension.^49^

## Sources of Funding

R.W. Hunter is supported by the Wellcome Trust-University of Edinburgh Institutional Strategic Support Fund. N. Dhaun is supported by a British Heart Foundation (BHF) Intermediate Clinical Research Fellowship (FS/13/30/29994). The authors also acknowledge funding from the BHF CoRE.

## Disclosures

None.

## Supplementary Material

## Novelty and Significance

### What Is New?

- This is the first study to explore the effects of big endothelin-1 (ET-1) on renal sodium and water handling in man.
- Our study protocol allowed us to determine the effects of big ET-1 on the kidney without significant hemodynamic perturbation.

### What Is Relevant?

- Big ET-1 infusion elicited a clinically significant natriuresis and diuresis.
- Renal clearance data and a molecular analysis of sodium transporter expression in urinary extracellular vesicles suggest that big ET-1 inhibits sodium reabsorption through ENaC in the collecting ducts.
- Endothelin receptor antagonists have been associated with edema and heart failure in clinical trials. Our results suggest this adverse effect is a consequence of stimulated sodium reabsorption in the collecting ducts.

### Summary

In healthy, infusion of big ET-1 elicited a natriuresis (via inhibition of sodium reabsorption in the renal collecting ducts). Blockade of this pathway may account for the edema and heart failure observed with the clinical use of endothelin receptor antagonists. Our data support the use of potassium-sparing diuretics as a therapeutic strategy to ameliorate fluid retention associated with these agents.
