# Supplementary material for: First-in-Man Demonstration of Direct Endothelin-Mediated Natriuresis and Diuresis
Source: Hypertension. 2017 May 30;70(1):192–200. doi: 10.1161/HYPERTENSIONAHA.116.08832 (PMC5739104; doi:10.1161/HYPERTENSIONAHA.116.08832)
Supplement: Supplementary file 1 [file hyp-70-192-s001.docx]

# Online data supplement

**Pregnancy outcome after first trimester use of methyldopa: A prospective observational cohort study**

Maria Hoeltzenbein^1^, Evelin Beck^1^, Anne-Katrin Fietz^1, 2^, Juliane Wernicke^1^, Sandra Zinke^1^, Angela Kayser^1^, Stephanie Padberg^1^, Corinna Weber-Schoendorfer^1^, Reinhard Meister^2^, Christof Schaefer^1^

^1^ Pharmakovigilanzzentrum Embryonaltoxikologie, Institut für Klinische Pharmakologie und Toxikologie, Charité - Universitätsmedizin Berlin

^2^ Department of Mathematics, Beuth Hochschule für Technik Berlin (University of Applied Sciences), Berlin, Germany

Running title: First Trimester Use of Methyldopa

Corresponding author:

Dr. med. Maria Hoeltzenbein

Pharmakovigilanzzentrum Embryonaltoxikologie

Institut für Klinische Pharmakologie und Toxikologie,

Charité - Universitätsmedizin Berlin

Augustenburger Platz 1

D - 13353 Berlin

Germany

Tel.: +49 (0) 30 450 525 702

Fax: +49 (0) 30 450 525 902

maria.hoeltzenbein@charite.de

# Supplementary Tables

Table S1. Exclusion criteria for both cohorts:

| Established teratogens | Retinoids (acitretin, adapalen, isotretinoin, tazaroten, tretinoin), carbamazepine, lenalidomide, methotrexate, mycophenolate, phenobarbital, phenprocoumon, phenytoin, thalidomide, topiramate, valproate, warfarin. |
| --- | --- |
| Fetotoxic drugs | Angiotensin-converting-enzyme-inhibitors and angiotensin II-receptor blockers. |
| Treatment indication | Malignancies and malignancy related conditions. |

Table S2. Maternal baseline characteristics and obstetric history.

| **Maternal characteristics** | **Methyldopa cohort** | **Comparison cohort** |
| --- | --- | --- |
|  | n=261 | n=526 |
| **Age, n** | 261 | 525 |
| Age (yrs) | 33 (30-37) (18-49) | 32 (28-35) (16-44) |
| **BMI, n** | 238 | 457 |
| BMI (kg/m^2^) | 27.8 (23.5-34.8) (18.4-58.8) | 22.5 (20.6-25.7) (16.8-51.6) |
| **Educational level, n** | 151 | 273 |
| no leaving exam | 4 (2.6) | 1 (0.4) |
| 9 years exam | 9 (6) | 14 (5.1) |
| 10/11 years exam | 55 (36.4) | 78 (28.6) |
| secondary school exam | 33 (21.9) | 64 (23.4) |
| academic study | 50 (33.1) | 116 (42.5) |
| **Smoking, n** | 258 | 519 |
| No | 238 (92.2) | 431 (83) |
| <= 5 cig/day | 5 (1.9) | 27 (5.2) |
| > 5 cig/day | 15 (5.8) | 61 (11.8) |
| **Alcohol, n** | 259 | 519 |
| No | 249 (96.1) | 485 (93.4) |
| <= 1 drink/day | 7 (2.7) | 22 (4.2) |
| > 1 drink/day | 3 (1.2) | 12 (2.3) |
| **Social drugs, n** | 244 | 505 |
| Yes | 0 (0) | 9 (1.8) |
| No | 244 (100) | 496 (98.2) |
| **Pregnancy wanted, n** | 219 | 429 |
| Yes | 210 (95.9) | 393 (91.6) |
| Indifferent | 8 (3.7) | 28 (6.5) |
| No | 1 (0.5) | 8 (1.9) |
| **Previous pregnancies, n** | 260 | 521 |
| 0 | 98 (37.7) | 211 (40.5) |
| 1 | 84 (32.3) | 174 (33.4) |
| 2 | 40 (15.4) | 81 (15.5) |
| 3 or more | 38 (14.6) | 55 (10.6) |
| **Previous deliveries, n** | 260 | 522 |
| 0 | 131 (50.4) | 268 (51.3) |
| 1 | 90 (34.6) | 177 (33.9) |
| 2 | 26 (10) | 57 (10.9) |
| 3 or more | 13 (5) | 20 (3.8) |
| **Previous miscarriages, n** | 260 | 519 |
| 0 | 197 (75.8) | 427 (82.3) |
| 1 | 43 (16.5) | 66 (12.7) |
| 2 or more | 20 (7.7) | 26 (5) |
| **Previous elective terminations, n** | 260 | 519 |
| 0 | 240 (92.3) | 490 (94.4) |
| 1 | 18 (6.9) | 24 (4.6) |
| 2 or more | 2 (0.8) | 5 (1) |
| **Previous children with anomalies, n** | 259 | 518 |
| 0 | 250 (96.5) | 505 (97.5) |
| 1 | 7 (2.7) | 13 (2.5) |
| 2 or more | 2 (0.8) | 0 (0) |
| **Gestational week at enrollment , n** | 261 | 526 |
| Week at first enrollment | 9.4 (6.6-15.1) (0-39.1) | 9 (6.4-14.9) (0-40) |

For age and gestational week at enrollment, median, interquartile range, and min/max are presented; n, number of informative cases (%); BMI, body mass index.

Table S3. Exposure details of antihypertensive treatment in the methyldopa cohort.

| Methyldopa dose (mg), n= 232 | 500 (250-1000, 125-3000) |
| --- | --- |
| Treatment duration (days), n= 258 | 199.4 (225.5, 177.2-264.8, 1-294) |
| **Initiation of methyldopa treatment**, n= 259 |  |
| Before conception | 140 (54%) |
| During first trimester | 119 (46%) |
| **Methyldopa monotherapy** | 127 |
| **Concomitant first trimester exposure to beta-blockers***, n=261 |  |
| Metoprolol | 71 |
| Bisopolol | 28 |
| Other betablockers | 8 |
| **Concomitant first trimester exposure to other antihypertensives***, n=261 |  |
| Calcium-channel blockers | 32 |
| Diuretics (hydrochlorothiazide or furosemide) | 12 |
| Other antihypertensives | 15 |

Median, interquartile range, and min/max for dose and treatment duration are presented;
n, number of informative cases.

*More than one co-medication in one patient is possible, only treatment with antihypertensive drugs is presented.

Table S4. Major birth defects and late fetal losses in prospectively ascertained pregnancies exposed to methyldopa.

| **Study no.** | **GW at call** | **Methyldopa exposure** | | | | | **Co-medication (indication)**\|  **exposure time (from- to GW)** | **GW at outome (birth/**  **pregnancy loss)** | **Sex/birth weight [g]** | **Details of pregnancies with major malformation or late pregnancy losses** | **Maternal age**  **[yrs]** |
| --- | --- | --- | --- | --- | --- | --- | --- | --- | --- | --- | --- |
|  |  | **Start**  **[GW]** | **Stop [GW]** | **Trimester** | **Dose [mg/d]** | |  |  |  |  |  |
| #1 | 21+3 | prec. | 23+4 | 1.-2. | 2000 | | Sertraline (depression)\|0-24+4  Quetiapine (depression)\|0-24+4  Lorazepam (depression)\|n.a.  Diazepam (depression)\|21-n.a.  Nifedipine (hypertension)\|n.a. | 24+4 | m / 450 | Atresia of pulmonary valve PDA, VSD, ASD (perinatal death) | 36 |
| #2 | 28+1 | prec. | 29+6 | 1.-3. | 750 | | Nifedipine (hypertension)\|0-28  Mefenamic acid (migraine\|0-29  Pantoprazole (prophylaxis)\|0-29+6  Paracetamol (migraine\|29+6  Metoprolol (hypertension)\|0-29+6 | 29+6 | m/ 1680 | ASD | 32 |
| #3 | 11+4 | 5+0 | 38+6 | 1.-3. | 250 | | Omeprazole (gastroesophageal reflux)\|5-38+6  Estradiol (prophylaxis SAB) \|0-12  Progesterone (prophylaxis SAB)\|0-12  Penicillin (bacterial infection)\|20-24  Insulin (diabetes type II)\|5-38+6 | 38+6 | m / 3900 | VSD  Macrocephaly | 41 |
| #4 | 14+3 | n.a. | n.a. | 1.-3. | n.a. | | Metformin, rosiglitazon (diabetes type II)\|0-9  Insulin (diabetes type II)9-36+6  Antibiotics (bacterial infection)\|n.a. | 36+6 | m / 2770 | ASD | 39 |
| #5 | 27+0 | prec. | 38+2 | 1.-3. | 375 | | Hydrocortisone (empty sella)\| 0-38+2  Estradiol (prophylaxis SAB?)\|0-24  Progesterone (prophylaxis SAB)\|0-24  Desmopressin (empty sella)\|0-38+2  Levothyroxine (empty sella)\|0-38+2 | 38+2 | m / 3340 | Hypospadias (grade II) | 32 |
| #6 | 4+0 | prec. | 38+3 | 1.-3. | 750 | | Metoprolol (hypertension)\|0-38+3  Dihydralazine (hypertension)\|0-4  rednisolone (infertility)\|0-13  Acetylsalicylic acid (infertility)\|0-8  Cefuroxime (pneumonia)\|5-6  Fusidine (local bacterial infection)\|13-14  Salbutamol (allergy)\|5-15 | 38+3 | m / 2910 | Club foot (left) | 42 |
| #7 | 36+1 | prec. | 39+6 | 1.-3. | 1000 | | Nystatin (vaginal mycosis)\|n.a. | 39+6 | m / 2760 | Club foot (left), Naevus (left lower leg) | 36 |
| #8 | 24+0 | prec. | 28+0 | 1.-3. | 500 | | Metoprolol (hypertension)\|0-28  Amlodipine (hypertension)\|10-28  Levothyroxine (hypothyroidism)\|0-28  Tacrolimus (renal transplant)\|0-28  Azathioprine (renal transplant)\|0-28  Betamethasone (fetal lung maturation)\|25+28  Ampicilline (bacterial infection)\|28  Nifedipine (tocolysis)\|25+28  Fenoterol (tocolysis)\|28 | 28+0 | m / 2340 | Congenital cystic adenomatous lung malformation,  Hydrops fetalis  (death because of respiratory insufficiency) | 33 |
| #9 | 6+0 | 5+6 | 6+3 | 1. | 250 | | Enoxaparin (thrombophilia)\|5-21  Acetylsalicylic acid (thrombophilia)\|21-37  Ofloxacin (dacryocystitis)\|6-7  Erythromycin (dacryocystitis)\|6-7  Metoprolol (hypertension)\|0-6 | 41+2 | m / 3995 | Hydronephrosis | 33 |
| Stillbirth and late fetal losses | | | | | | | | | | | |
| #10 | 10+0 | 9+1 | 38+5 | 1.-3. | 2000 | Lercanidipine (hypertension)\|5-9  Bisoprolol (hypertension)\|0-38+5  Insulin human (gestational diabetes)\|25-38+5  Acetylsalicylic acid (low dose, prophylaxis)\|23-34 | | 38+5 | m / 2790 | Stillbirth (nuchal cord) | 32 |
| #11 | 8+0 | 8 | 16 | 1.-2. | 250 | Nebivolol (hypertension)\|0-8 | | 16+0 | ? | Spontaneous abortion | 40 |
| #12 | 17+0 | 11 | 17-? | 1.-2. | 1500 | Acetylsalicylic acid (cardiac insufficiency)\|11-17-?  Amoxicilline (urinary tract infection)\|11-15  Furosemide (cardiac insufficiency\|17-18  Insulin (diabetes type I)\|0-17-?  Metoprolol (hypertension)\|11-17-?  Dihydralazine (hypertension)\|11-17-?  Hydrochlorothiazide (hypertension)\|11-17-? | | 20+0 | ? | Fetal loss (premature rupture of membranes) | 42 |

Abbreviations:

ASD, Atrial septal defect; VSD, ventricular septal defect; PDA, persistent ductus arteriosus Botalli; SAB, spontaneous abortion; prec., preconception; n.a., not applicable; f, female; m, male; GW, gestational week

Table S5. Adjusted standard deviation scores (SDS_adj_) for head circumference and birth weight of methyldopa exposed neonates.

| **Outcome** | **Overall** | **Male** | **Female** |
| --- | --- | --- | --- |
|  | SDS_adj_ difference  (95% CI) | SDS_adj_ difference  (95% CI) | SDS_adj_ difference  (95% CI) |
| Birth weight | -0.34 (-0.54 to -0.14) | -0.30 (-0.59 to -0.02) | -0.38 (-0.66 to-0.1) |
| Head circumference | -0.24 (-0.46 to -0.03) | -0.32 (-0.61 to -0.03) | -0.16 (-0.46 to 0.15) |

# Supplementary Figures

Figure S1.


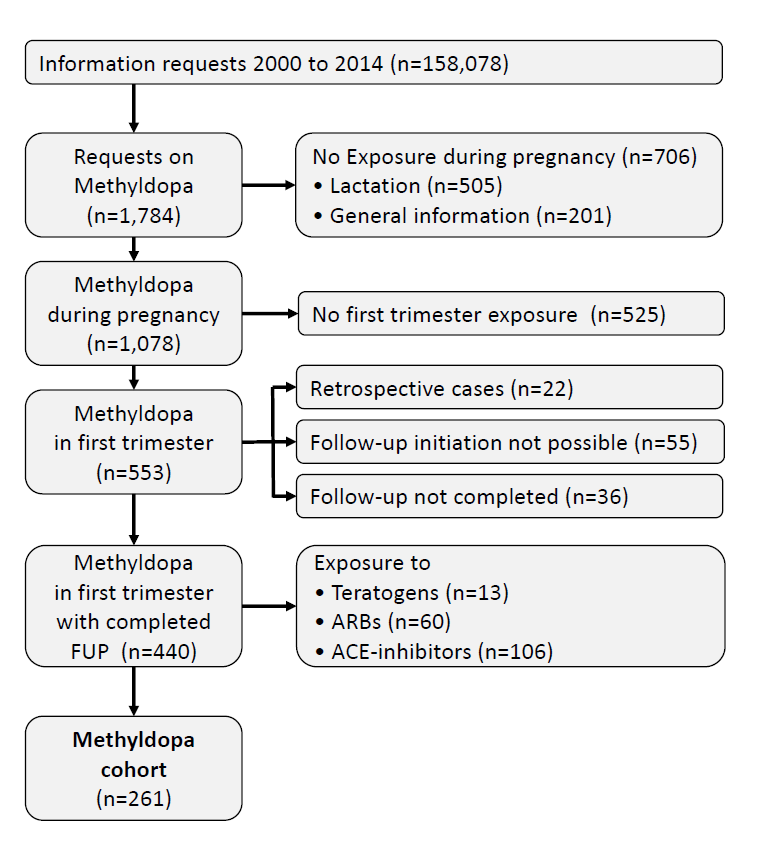


Figure S1. Overview on information requests on methyldopa to the German Embryotox pharmacovigilance institute from January 1, 2000, to December 31, 2014. n, number of requests; FUP follow-up, ACE, angiotensin-converting enzyme; ARBs angiotensin II-receptor-antagonists.

Figure S2.


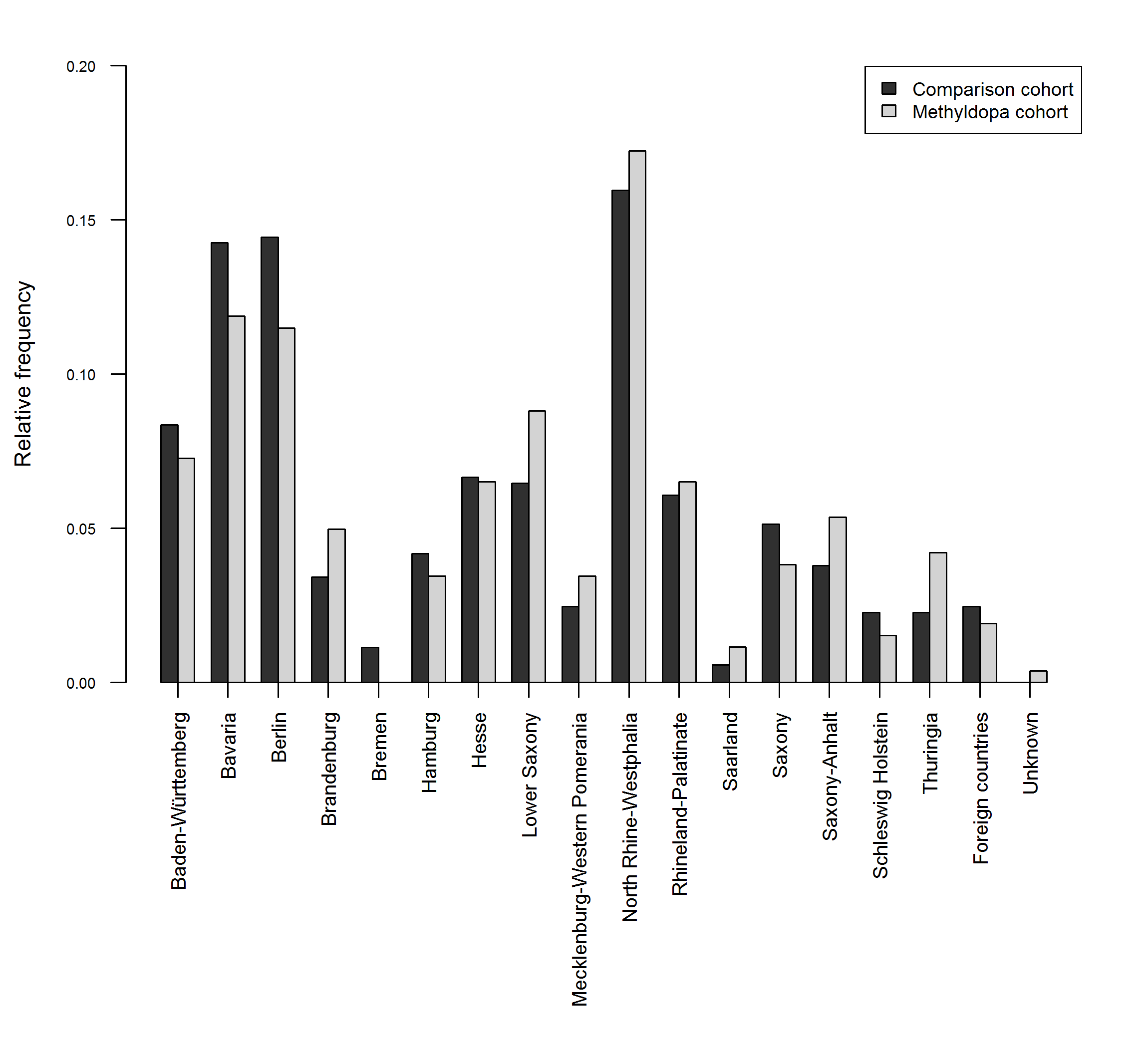


Figure S2: Distribution of federal states of Germany within methyldopa and comparison cohort. Relative frequencies correspond to the number of inhabitants of the respective federal state, except for Berlin, which is overrepresented, as known from analyzing all enquiries to our institute.

Figure S3.


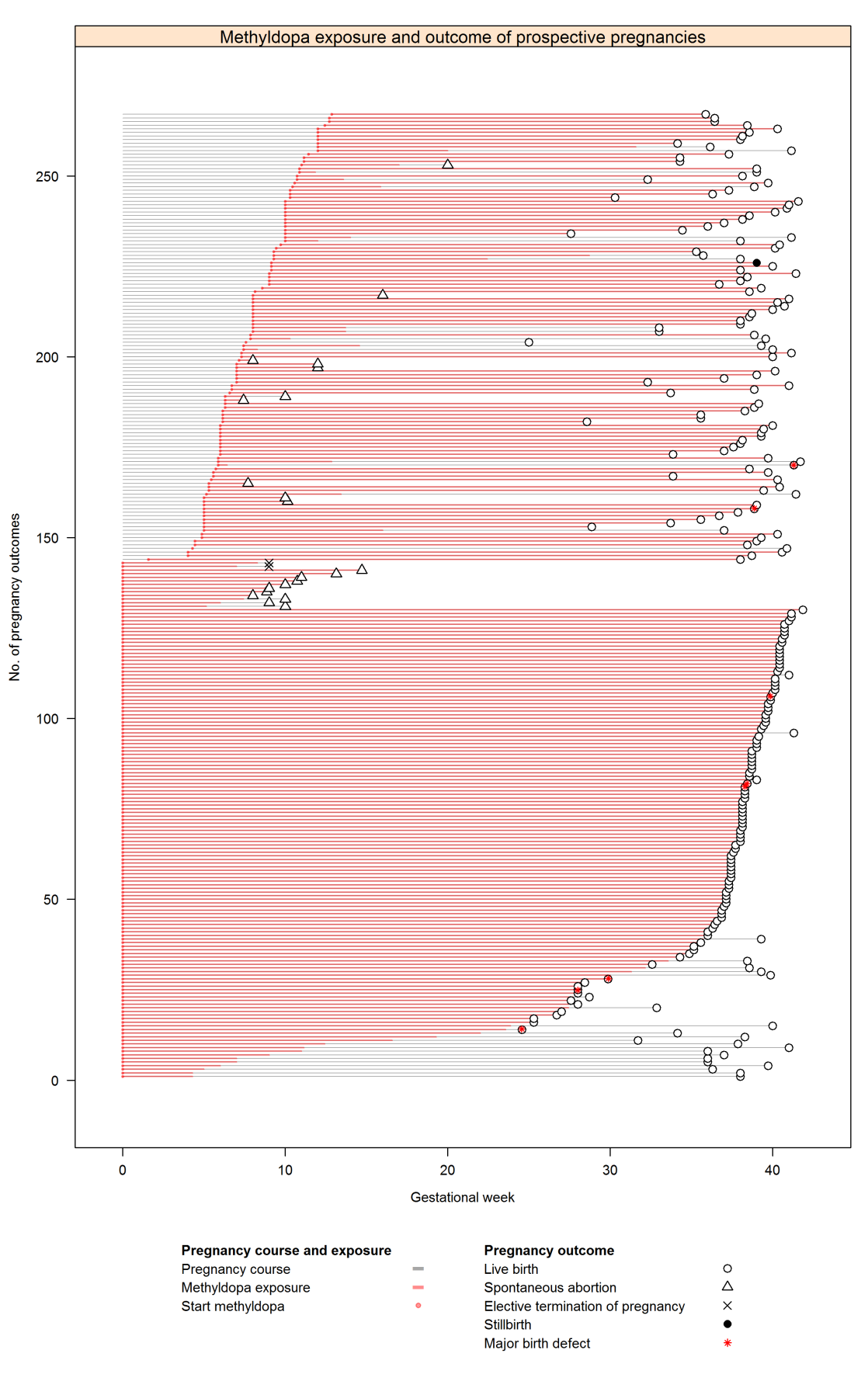


Figure S3. Course and outcome of exposed pregnancies (n=258) with each line representing one pregnancy, multiple pregnancies are represented by two lines. The graphic includes start and stop of methyldopa, as well as major birth defects. Only pregnancies with complete information on exposure are represented.

Figure S4.


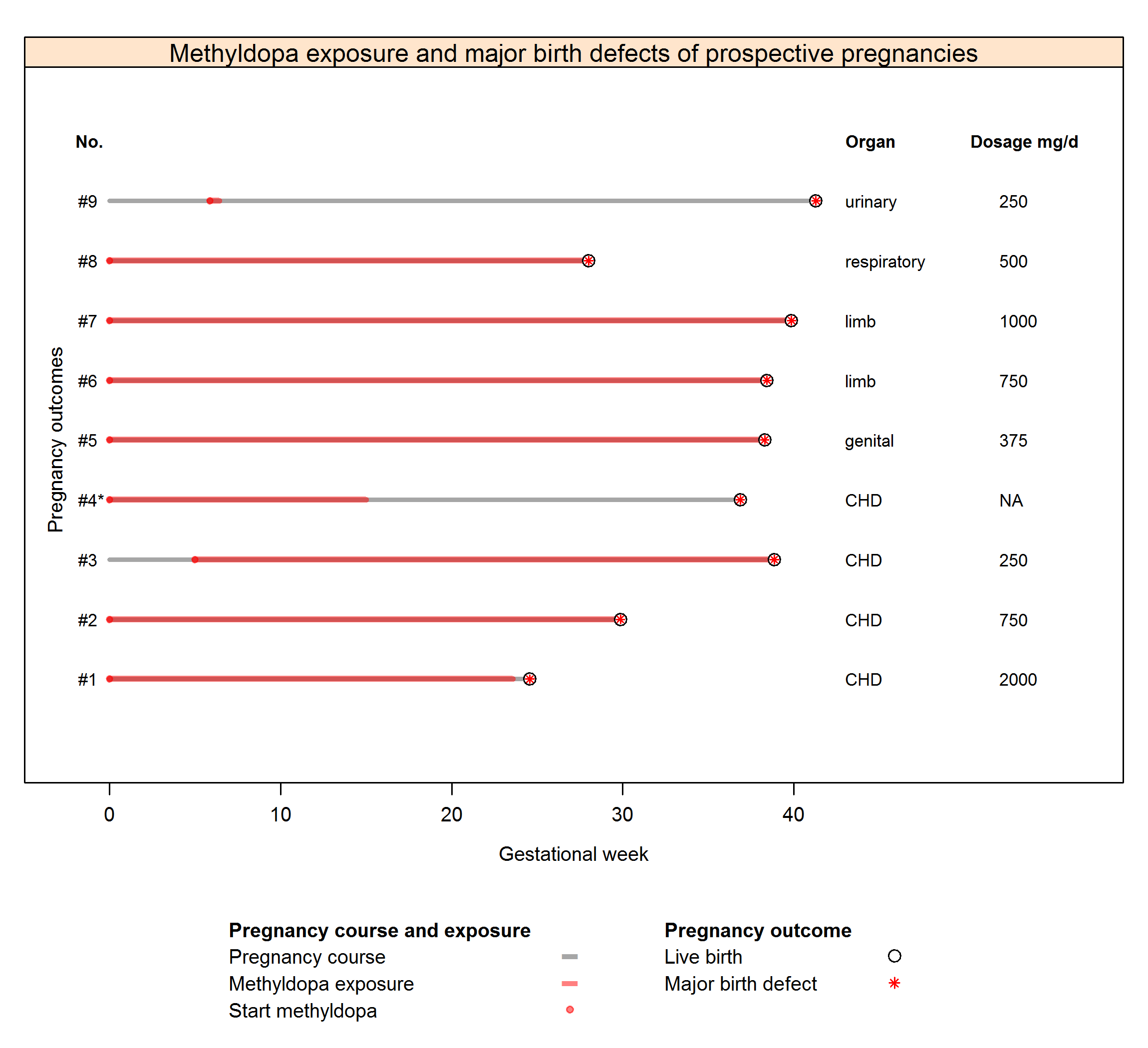


Figure S4. Exposure characteristics and course of pregnancy in prospectively ascertained pregnancies with major birth defects, with one line corresponding to each affected pregnancy (n=9). The graphic includes start and stop of methyldopa, daily dose, gestational week at birth and the organ system affected by major birth defects.
CHD, congenital heart defect
*****Exposure status unknown after week 15.

Figure S5a.


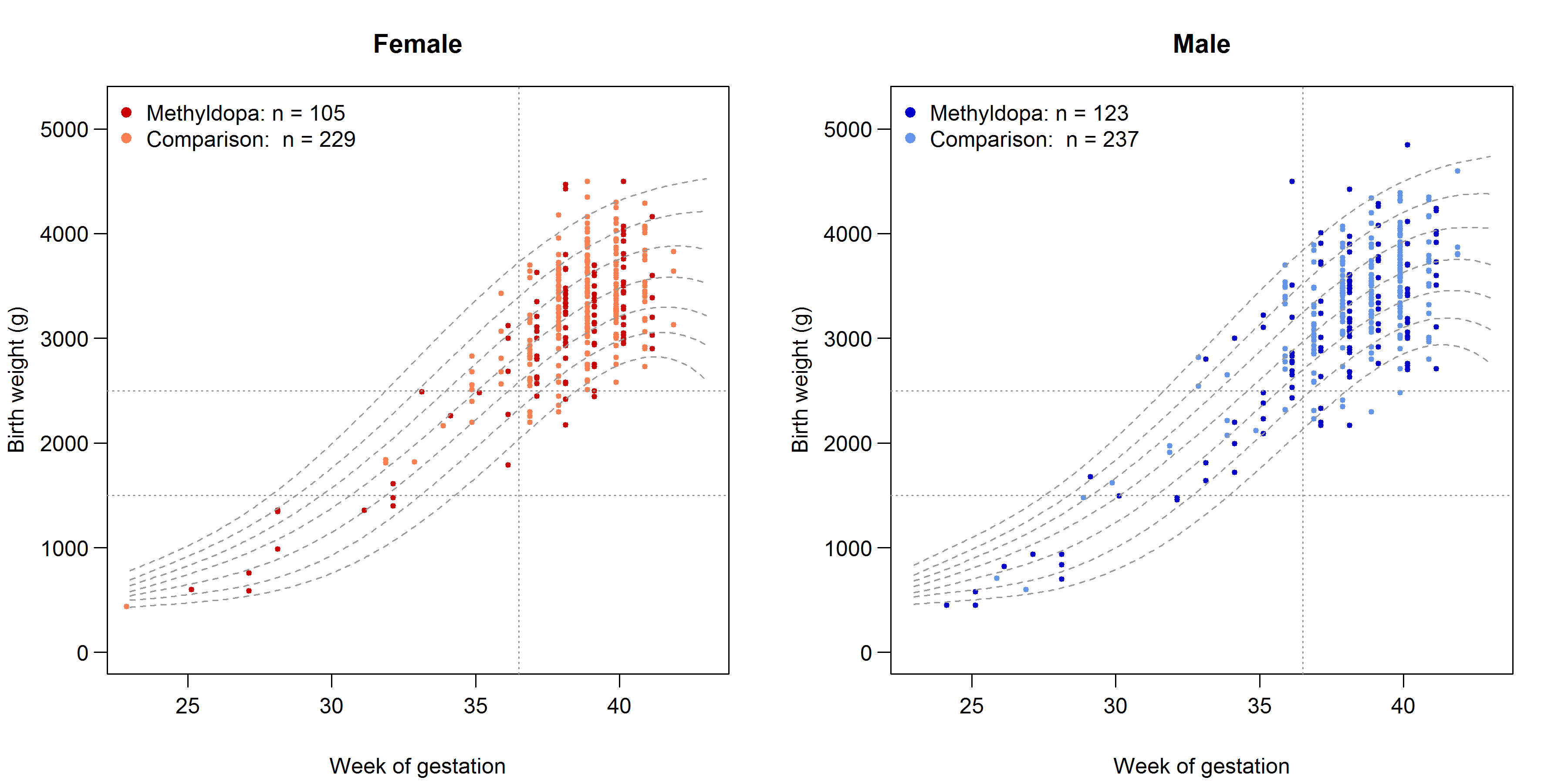


Figure S5b.


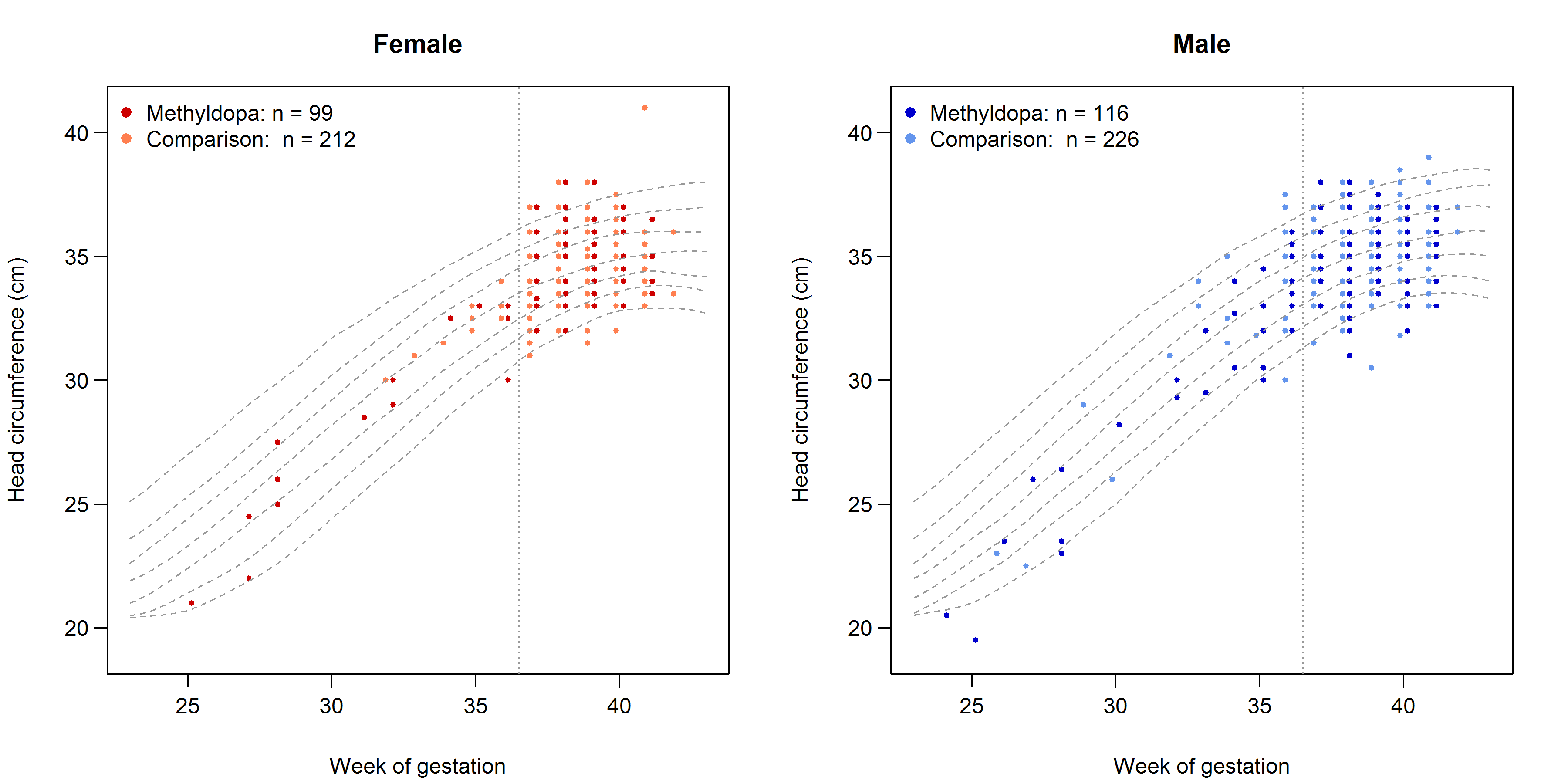


Figure S5. Birth weight (a) and head circumference (b) in relation to gestational age based on the German perinatal survey for neonates of the methyldopa and comparison cohort. Smoothed centile curves for birth weight according to gestational age (3rd, 10th, 50th, 90th, and 97th), only those neonates with complete information on birth weight, gestational age at birth and sex are shown.
